# Dynamical Simulations of Carotenoid Photoexcited States Using Density Matrix Renormalization Group Techniques

**DOI:** 10.1021/acs.jpca.3c00988

**Published:** 2023-04-13

**Authors:** Dilhan Manawadu, Darren J. Valentine, William Barford

**Affiliations:** † Department of Chemistry, Physical and Theoretical Chemistry Laboratory, University of Oxford, Oxford OX1 3QZ, United Kingdom; ‡ Linacre College, University of Oxford, Oxford OX1 3JA, United Kingdom; ¶ Balliol College, University of Oxford, Oxford OX1 3BJ, United Kingdom

## Abstract

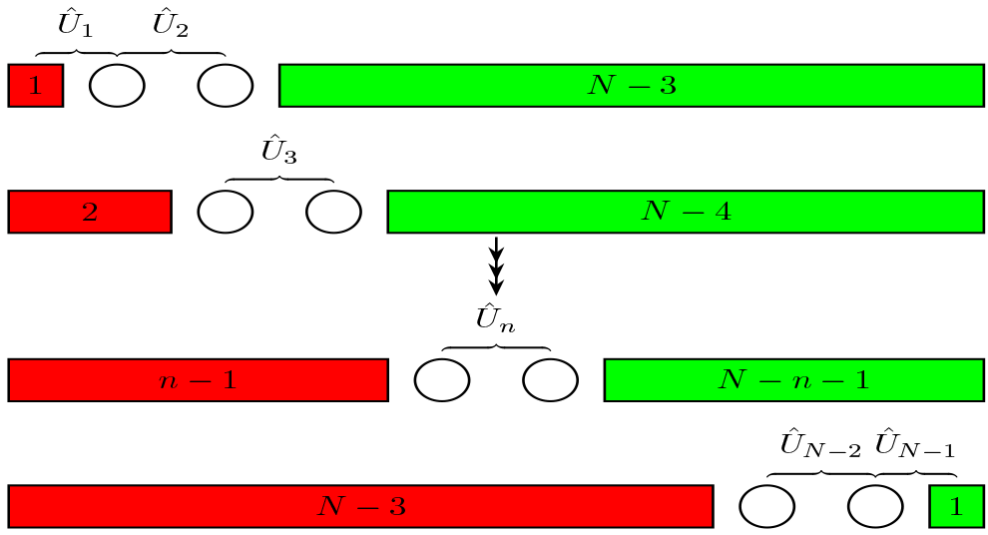

We present a dynamical simulation scheme to model the highly correlated excited state dynamics of linear polyenes. We apply it to investigate the internal conversion processes of carotenoids following their photoexcitation. We use the extended Hubbard-Peierls model, , to describe the π-electronic system coupled to nuclear degrees of freedom. This is supplemented by a Hamiltonian, , that explicitly breaks both the particle-hole and two-fold rotation symmetries of idealized carotenoid structures. The electronic degrees of freedom are treated quantum mechanically by solving the time-dependent Schrödinger equation using the adaptive time-dependent DMRG (tDMRG) method, while nuclear dynamics are treated via the Ehrenfest equations of motion. By defining adiabatic excited states as the eigenstates of the full Hamiltonian, , and diabatic excited states as eigenstates of , we present a computational framework to monitor the internal conversion process from the initial photoexcited 1^1^B_u_^+^ state to the singlet triplet-pair states of carotenoids. We further incorporate Lanczos-DMRG to the tDMRG-Ehrenfest method to calculate transient absorption spectra from the evolving photoexcited state. We describe in detail the accuracy and convergence criteria for DMRG, and show that this method accurately describes the dynamical processes of carotenoid excited states. We also discuss the effect of the symmetry-breaking term, , on the internal conversion process, and show that its effect on the extent of internal conversion can be described by a Landau–Zener-type transition. This methodological paper is a companion to our more explanatory discussion of carotenoid excited state dynamics in Manawadu, D.; Georges, T. N.; Barford, W. Photoexcited State Dynamics and Singlet Fission in Carotenoids. *J. Phys. Chem. A***2023**, *127*, 1342.

## 1 Introduction

Carotenoids are one of the most abundant classes of pigments found in nature with over 1100 distinct structures reported.^1^ Carotenoids are produced naturally by all photosynthetic organisms (including plants and algae) and some non-photosynthetic organisms.^2^ In physiological systems, they have varied functions including enhancing vision by quenching singlet oxygen and other radicals,^3^ serving as an antioxidant by scavenging cation radicals,^4^ enhancing immune functions,^5^ and acting as anticancer agents.^6^

**Figure 1:**
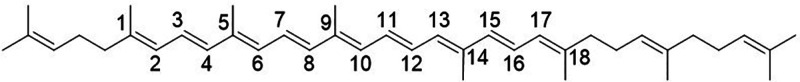
Neurosporene, a naturally occurring carotenoid with 18 conjugated carbon atoms.

As shown in Figure 1, carotenoids possess a conjugated polyene backbone of usually between 18 and 26 carbon atoms (or 9 to 13 C–C double bonds), which gives rise to their bright yellow, orange, and red colors due to the strong absorption of visible light in the blue-green region.^7^ This region is not accessible to chlorophylls, and therefore, carotenoids found in photosynthetic systems can enhance their light harvesting properties and protect the light harvesting (chlorophyll) complexes from excess light.^8−10^ Due to these crucial functions in photosynthetic systems, the study of carotenoid photoexcited state properties has gained considerable attention. Study of the photophysics of carotenoids is also motivated by their potential to be utilized as efficient singlet fission materials in photovoltaic devices. Singlet fission is a photophysical process by which a singlet photoexcited state dissociates into two spin uncorrelated triplets.^11^ Coupling of singlet fission materials to low-gap-semiconductors is known to increase the efficiency of photovoltaic devices and therefore is a major interest in the search for efficient renewable energy sources.^12^

As the polyene backbone of carotenoids gives rise to their photophysical features, theoretical studies model the carotenoid excited states by investigating the excited state properties of the related polyenes. Theoretical studies of the exotic nature of polyene excited states were pioneered by a seminal paper from Schulten and Karplus, which described the experimentally observed low-lying dark excited state of polyenes.^13,14^ They described the conjugated π-electron system using the semiempirical Pariser–Parr–Pople (PPP) Hamiltonian^15^ and the configuration interaction (CI) method description of the wave function with double excitations. Their work was followed by several studies based on semiempirical Hamiltonians, which helped formulate the theoretical understanding of polyene excited states.^16−22^

Early ab initio calculations of polyene excited states were based on self-consistent field (SCF) and CI calculations.^23,24^ Improvements to the ground and excited state geometries of polyenes were brought about by the use of the multiconfiguration self-consistent field (MCSCF) method.^25^ Serrano-Andres and co-workers introduced the second order perturbation theory method CASPT2 with a complete active space SCF (CASSCF) wave function as the reference state to study electronic states of polyenes.^26^ Their calculation provided the first evidence from an ab initio study for the existence of a dark low-lying polyene excited state. More recently, time-dependent density functional theory (TD-DFT),^27,28^ extended algebraic diagrammatic construction (extended-ADC(2)),^29^ CASSCF with *n*-electron valence perturbation theory (NEVPT),^30^ and density functional theory with a multireference configuration interaction^31,32^ have been utilized to study polyene excited states. However, application of complete active space methods to model long chain polyene systems is challenging because of the exponential growth of the many-body Hilbert space with the size of the single particle basis.

In 1992, White introduced the density matrix renormalization group (DMRG) algorithm to study strongly correlated quantum lattice systems.^33^ DMRG was soon adapted to study π-conjugated polymers^34−37^ and polyene photophysics;^38−43^ as applied to one-dimensional systems, it yields arbitrarily exact results for a finite-size Hilbert space. DMRG was first utilized to model ab initio Hamiltonians in 1999.^44^ Ghosh et al. demonstrated that the CASSCF method can be incorporated to a DMRG algorithm, allowing for modeling polyene systems of natural carotenoid lengths.^45^ While the inherent multireference nature of the DMRG accounts for static correlations, perturbative theory corrections are required to accurately describe the dynamic correlations present in the system.^46,47^ More recent ab initio studies on polyene excited states using multireference perturbation theory (MRPT) DMRG-CASSCF, which accounts for the dynamic correlations, have reignited the debate on carotenoid excited state energy ordering.^48,49^

The original DMRG formulation of White has been extended to form a family of time-dependent DMRG (TD-DMRG) algorithms designed to model time dependent phenomena of molecular systems. One of the widely used TD-DMRG algorithms is the adaptive time-dependent DMRG (tDMRG) algorithm, independently developed by Daley et al.,^50^ and White and Feiguin^51^ to study time evolution of weakly entangled systems. Examples of applications of adaptive tDMRG in molecular physics include modeling magnetization transport in spin-1/2 chains,^52^ demonstrating spin-charge separation in cold Fermi gases,^53^ calculating zero temperature conductance of strongly correlated nanostructures,^54^ elucidating transport properties of quantum-dot systems connected to metallic leads,^55^ evaluating spectral functions of spin-1 Heisenberg antiferromagnetic chain,^56^ exciton transport in one-dimensional Hubbard insulators,^57^ and non-equilibrium transport in single-impurity Anderson model.^58^ Techniques based on tensor network models have recently been used to study the dynamics of photophysical systems, for example, ultrafast relaxation and localization of photoexcited states in light emitting polymers,^59−61^ internal conversion in pyrazine,^62^ and singlet fission in substituted pentacene dimers.^63^ Readers are referred to a recent review by Ren and co-workers on applications of different TD-DMRG algorithms to model the dynamics of quantum systems.^64^

While state-of-the-art ab initio methods have had great success in calculating static properties of polyene excited states, due to computational expediencies the use of semiempirical Hamiltonians with a single electron basis is more attractive to model dynamical processes of photoexcited polyenes. With the correct parametrization, DMRG has been shown to work very well for semiempirical Hamiltonians with a reduced single-particle basis.^65−67^ The DMRG algorithm with the PPP Hamiltonian has been widely used to study the electronic properties of conjugated polyenes.^36,39−42,68,69^

Recently, the authors developed DMRG methods to simulate internal conversion of photoexcited states and singlet triplet-pair production in carotenoid systems of up to 22 conjugated carbon atoms. Carotenoids are particularly challenging as they exhibit strong electronic correlation and strong electron–nuclear coupling. In dimers, they also exhibit singlet fission after photoexcitation. In ref (70), we implemented mixed quantum-classical dynamics, treating the electrons via adaptive tDMRG and the nuclei via Ehrenfest dynamics, to simulate internal conversion in zeaxanthin. In a companion paper,^71^ we extend those simulations to neurosporene, and we also describe our calculations of transient absorption.

The primary purpose of this article is to describe in more detail the methodology of the tDMRG-Ehrenfest simulation for a wider theoretical chemistry community. In particular, we emphasize that tDMRG is a rather natural generalization of the finite-lattice algorithm of static DMRG. We also explain the Lanczos-DMRG method for computing the transient absorption spectrum of the time-evolving photoexcited state. We show that DMRG methods can accurately and reliably describe the complex photoexcited state dynamics of large linear conjugated systems.

In this paper, we also explore in more detail the excited state dynamics as a function of the broken-symmetry perturbation that connects the diabatic states of opposite particle-hole symmetry. We show that for a small perturbation, the system undergoes a transition from the initial adiabatic state, *S*_1_, to *S*_2_, while remaining in the same diabatic state, 1^1^B_u_^+^. In contrast, for larger perturbations, the system evolves adiabatically on the *S*_1_ surface, changing character from the excitonic 1^1^B_u_^+^ state to the singlet triplet-pair 2^1^A_g_^–^ state. In all cases, the *S*_1_ and *S*_2_ energies exhibit an avoided crossing, and the dynamics can be approximately modeled as a two-level system.

## 2 Model Hamiltonian and Eigenstates

### 2.1 UV-Peierls Hamiltonian

The PPP Hamiltonian, routinely utilized with static DMRG for modeling electronic properties of conjugated polymers, contains long-range Coulomb interactions and therefore is not readily suitable for dynamical simulations using the adaptive time-dependent DMRG (tDMRG) algorithm. In a tDMRG setting, the electronic degrees of freedom are conveniently described by the extended Hubbard (UV) Hamiltonian, defined by
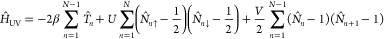
1where *n* labels the *n*^th^ C atom, *N* is the number of conjugated C atoms and *N*/2 is the number of double-bonds.  is the bond order operator,  creates (destroys) an electron with spin σ in the *p*_*z*_ orbital of the *n*^th^ C atom, and  is the number operator. *U* and *V* correspond to Coulomb parameters which describe interactions of two electrons in the same orbital and nearest neighbors, respectively, and β represents the electron hopping integral between neighboring C atoms. As described in ref (43),  can be derived from the Born–Oppenheimer Hamiltonian for the π-electrons.

With the inclusion of nuclear degrees of freedom, the UV-Peierls Hamiltonian is defined by
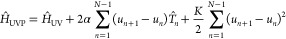
2where α is the electron–nuclear parameter, *K* is the nuclear spring constant, and *u*_*n*_ is the displacement of the *n*th carbon atom from its undistorted geometry. Electron hopping integrals relate to nuclear geometries via
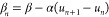
3In principle, the nearest-neighbor Coulomb interaction, *V*, also depends on the nuclear coordinates. However, Barford et al.^41^ showed that compared to the coupling to the bond-order operator this dependency has a negligible effect on the excited state energies of polyenes, and so we have chosen to neglect it.

The UV-Peierls Hamiltonian is invariant with a particle-hole transformation. For idealized carotenoid structures with *C*_2_ symmetry, its eigenstates will have definite *C*_2_ and particle-hole symmetries.^43^

### 2.2 Broken Symmetry

In order to facilitate internal conversion from the photoexcited 1^1^B_u_^+^ state to the triplet-pair states (which are of negative particle-hole character), an interaction term which breaks the particle-hole symmetry is introduced. (We follow the particle-hole sign convention of ref (72), which is commonly used by the experimental community but is the opposite definition to refs (41) and (43).) The symmetry-breaking term is defined by
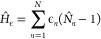
4where ϵ_*n*_ is the on-site potential energy parameter on the *n*^th^ C atom. The inclusion of *H*_ϵ_ is justified as carotenoids with both electron donating and withdrawing substituent groups (e.g., neurosporene shown in Figure 1), which act as electron donors and acceptors to the π-system and are known to undergo singlet fission.^7^

Since we are only interested in the singlet excitations, we project out the high spin contributions to the Hilbert space by supplementing the Hamiltonian with
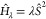
5where Ŝ is the total spin operator, and λ > 0.

We now define the full Born–Oppenheimer Hamiltonian as
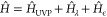
6Eigenstates of *Ĥ* are labeled adiabatic (singlet) eigenstates and are defined by
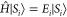
7We define a diabatic (singlet) basis spanned by the eigenstates of  as
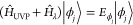
8The diabatic eigenstates have definite *C*_2_ and particle-hole symmetries.

We calculate the probability that the system described by Ψ(*t*) occupies the adiabatic state *S*_*i*_ by
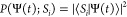
9and the probability that it occupies the diabatic state ϕ_*j*_ by
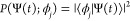
10Finally, the probabililty that the adiabat *S*_*i*_ occupies the diabat ϕ_*j*_ is
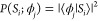
11

Diabatic eigenstates of  with a positive particle-hole symmetry are termed “ionic”, as the expectation value of the ionicity operator, , is larger for these states than for eigenstates with a negative particle-hole symmetry, termed “covalent”. We use this property of a larger ionicity for ionic states to distinguish them from covalent states during the dynamical simulation.

### 2.3 Parametrizations

#### 2.3.1 UVP Model Parametrization

The UV Hamiltonian does not contain the long-range Coulomb interactions of the PPP Hamiltonian and therefore requires a parametrization of the *U* and *V* Coulomb parameters to replicate the PPP model predictions. While retaining the Chandross-Mazumdar parametrization of β = 2.4 eV,^65^*K* = 46 eV Å^–2^ and α = 4.593 eV Å^–1^ from Barford and co-workers,^41^ in our earlier work^70^ we parametrized the UV model for internal conversion from 1^1^B_u_^+^ to 1^1^B_u_^–^ to reproduce the predictions of ref (72), which is a PPP model calculation. Reference (72) reports on DMRG calculations of carotenoids using the well-parametrized PPP model (i.e., with long-range Coulomb interactions), so it is important that the UV model reproduces those calculations.

In our companion paper,^71^ we model internal conversion from the 1^1^B_u_^+^ state to the 1^1^B_u_^–^ and 2^1^A_g_^–^ states. For the latter, we require a parametrization where *E*_1^1^B_u_^+^_ (vertical) < *E*_2^1^A_g_^–^_ (vertical). This pathway is parametrized using Taffet et al.,^48^ which reports highly accurate ab initio calculations on a series of carotenoids related to our work. They used DFT, TDDFT, and CASSCF(4,4) to determine the geometric minima of the 1^1^A_g_^–^, 1^1^B_u_^+^, and 2^1^A_g_^–^ states, respectively. Then they performed DMRG self-consistent-field (DMRG-CASSCF) calculations to determine the excitation energies, with N-electron valence-state perturbation theory (NEVPT2) correction to account for dynamic correlations.

For a given *U*, increasing *V* decreases . Keeping all other parameters the same (i.e., *U* = 7.25 eV and β = 2.4 eV), we find *V* = 3.25 eV such that (vertical) (vertical) replicates the lowest-lying carotenoid dark and bright state vertical excitation energies reported in Table 2 of ref (48). The diabatic vertical and relaxed energies for the UV-Peierls model with these parameters are illustrated in Figure S1 of ref (71). (The corresponding figures where (vertical) (vertical) for *U* = 7.25 eV, β = 2.4 eV, and *V* = 2.75 eV are shown in ref (70).) For all carotenoid chain lengths under consideration, 1^1^B_u_^+^ vertical energies lie below 2^1^A_g_^–^ vertical energies, while 1^1^B_u_^+^ relaxed energies are above 2^1^A_g_^–^ relaxed energies, indicating the possibility of internal conversion from the 1^1^B_u_^+^ to the 2^1^A_g_^–^ states via a diabatic energy level crossing.

#### Parametrizing 

2.3.2

The symmetry-breaking term (eq 4) alters the on-site potential energies and therefore changes the Mulliken charge densities of the π-system from unity. As outlined below, we use the ground state Mulliken charge densities of the π-system to parametrize *H*_ϵ_ for neurosporene, the structural formula of which is illustrated in Figure 1. The optimum Mulliken charge densities are calculated using the ORCA program package.^73,74^ Geometry optimizations are performed using density functional theory (DFT) with a B3LYP functional^75^ and a def2-TZVP basis set^76,77^ followed by calculations of electron densities. To enforce charge neutrality in our model, the mean shifted Mulliken charge densities are used as target densities in an optimization algorithm to determine .

Allowing unconstrained optimization of  leads to unphysical on-site potential energies and significant changes in the character of excited states. In order to avoid large perturbations, we use projected gradient descent algorithm to search for  such that |ϵ_*i*_| < ϵ_max_, *∀ i* ∈ {1, 2, ..., *N*}.^78^ For a given **ϵ**, ***d*** = (*d*_1_, *d*_2_, ..., *d*_*N*_) where  can be found via the static DMRG algorithm. We define the minimization function as *E*(**ϵ**) = ∥***d***_opt_ – ***d***(**ϵ**)∥, where ***d***_opt_ is the target density vector found via DFT. The algorithm is as follows:

1. Choose initial **ϵ**_0_ within the constraints
2. Loop until the convergence condition is met:
  (a) Find the descent direction – ∇*E*(**ϵ**_*k*_)
  (b) Find
  (c) Projection: Find  such that *∀ i* ∈ {1, 2, ..., *N*}

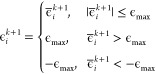
12

3. Convergence is evaluated via the coefficient of variation *r*^2^(**ϵ**) defined as

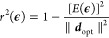
13

We perform the optimization for ϵ_max_ = 1.0 eV. For the simulations described in ref (71), ϵ_max_ is constrained to an upper bound of 1.0 eV to prevent the formation of an unphysical potential energy gradients across the conjugated carbon atoms which causes an unphysical mixing of the ionic and covalent states. (However, the effect of an arbitrary symmetry-breaking potential is described in section 7.2 of this paper.) The optimized  found for neurosporene with *V* = 3.25 eV and *r*^2^(**ϵ**) = 0.92 is shown in Table S1 of ref (71).

### 2.4 Initial Conditions

For our dynamical simulations, we first determine the ground state of the system using the static DMRG method by solving eq 2 for fixed nuclear displacements, {*u*_*n*_}. The Newtonian force acting on atom *n*, *f*_*n*_, is given by the Hellmann–Feynman theorem
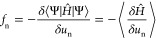
14Starting from the undimerized geometry (*u*_*n*_ = 0, ∀*n*), ground and excited state energies and geometries are found by iterative application of eq 14 with the force per atom *n*, *f*_n_ = 0.^41^ The initial system, Ψ(*t* = 0), is taken to be the adiabatic singlet state *S*_*i*_ with the largest projection onto the 1^1^B_u_^+^ diabat, determined via eq 11. This corresponds to a dipole-allowed vertical excitation from the ground state, *S*_0_. The choice to define the initial system this way, instead of as , is based on the observation that the Ehrenfest approximation (discussed in section 4) is most accurate for systems evolving on a single adiabatic potential energy surface.

## 3 Density Matrix Renormalization Group

From now on in this paper, we define a “site” as a p_*z*_ orbital of a C atom. The single-site basis for the UVP model defined in eq 2, i.e., {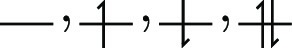}, has a dimensionality of 4. Therefore, exactly solving the time-dependent Schrödinger equation for *N* = 18, the relevant carotenoid chain length, would require solving a *S*_*z*_ = 0 Hilbert space of size ≈2.4 × 10^9^. This is not feasible on realistic time scales. DMRG methods are based on the premise that by an efficient truncation of the exact Hilbert space to retain only the important many-particle states, the most important features of the system can be preserved at a significantly lower computational cost. We begin this section by describing the static DMRG algorithm and then show how the method can easily be extended to the adaptive time-dependent DMRG (tDMRG) algorithm.

### 3.1 Static DMRG Algorithm

**Figure 2:**
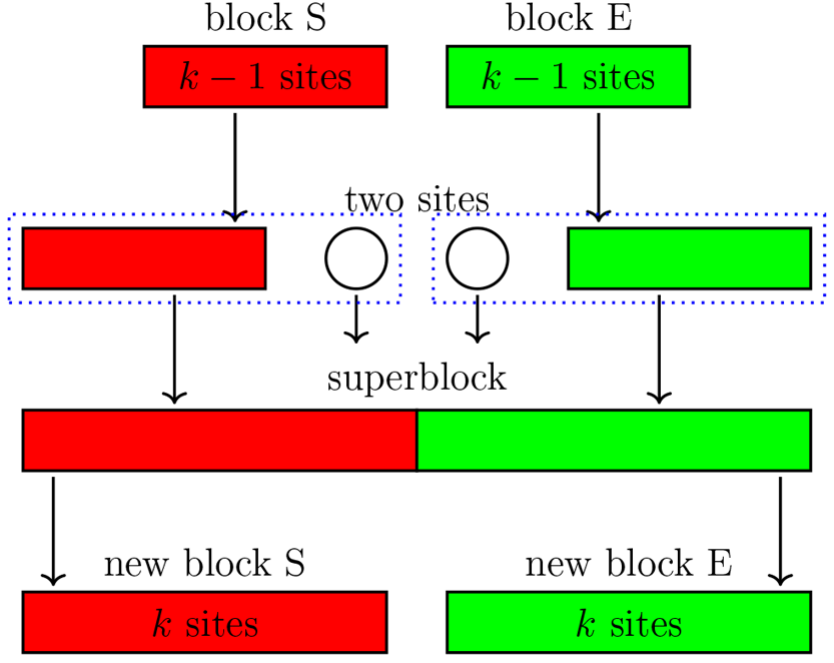
Schematic diagram illustrating one iteration of the infinite DMRG algorithm.^79^ The system block (S) is shown in red as the left-hand block, while the environment block (E) is shown in green as the right-hand block. Each site is an p_*z*_ orbital of a C atom.

The infinite DMRG algorithm was introduced in 1992 to accurately calculate ground states of 1D quantum systems.^33^ We begin by assuming that we have an accurate description of a physical system of length (2*k* – 2) sites as a product of two Hilbert spaces of physical length (*k* – 1) sites each. We denote these two constituent subsystems as a primitive system block (S) and primitive environment block (E), as shown in Figure 2. The primitive system block of length (*k* – 1), described by a Hilbert space of size *M*_*l*_ is spanned by the basis states {|*l*_*k*–1_⟩}. The primitive environment block of length (*k* – 1), described by a Hilbert space of size *M*_*r*_ is spanned by the basis states {|*r*_*k*–1_⟩}.

Consider the process of the linear growth of the system block by adding a single site at index *k*, as shown in Figure 2. The single site is fully described by the *d*-dimensional basis {|α_*k*_⟩}. An augmented system block of length *k* is constructed by combining the primitive system block and the site at index *k*. This augmented system block is described by the product Hilbert space spanned by {|*l*_*k*–1_⟩ ⊗ |α_*k*_⟩, with dimensions of *N*_*S*_ = *M*_*l*_ × *d*. An analogous augmented environment block is constructed by combining the site at index *k* + 1 and the primitive environment block. The augmented environment block is described by the product Hilbert space spanned by {|α_*k*+1_⟩ ⊗ |*r*_*k*–1_⟩, with dimensions *N*_*E*_ = *d* × *M*_*r*_. By combining the two augmented blocks, a superblock of length 2*k* is now formed in the product Hilbert space spanned by {|*l*_*k*–1_⟩ ⊗ |α_*k*_⟩ ⊗ |α_*k*+1_⟩ ⊗ |*r*_*k*–1_⟩}. The ground state
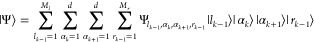
15is obtained by a sparse-matrix diagonalization (e.g., conjugate gradient or Davidson) of the Hamiltonian in the superblock basis.

Defining the augmented system block state |*i*⟩ ∈ {|*l*_*k*–1_⟩ ⊗ |α_*k*_⟩, and the augmented environment block state |*j*⟩ ∈ {|α_*k*+1_⟩ ⊗ |*r*_*k*–1_⟩, the ground state may also be expressed as
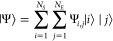
16Now a truncation procedure must be introduced to describe the system block of size *k* using a basis of dimension *M*_*S*_ < *N*_*S*_. Suppose that the ground state of the system can be expressed by the approximate state  in this truncated Hilbert space:
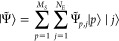
17Finding the optimum  is achieved by the minimization of the quadratic norm *S*_2_:
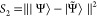
18

From eq 16, we see that Ψ can be recast into a rectangular matrix of dimension *N*_*S*_ × *N*_*E*_, which can then be decomposed using singular value decomposition as
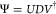
19where *U* is a unitary matrix of dimension *N*_*S*_ × *N*_*E*_, *V* is a unitary matrix of dimension *N*_*E*_ × *N*_*E*_, and *D* is a diagonal matrix of dimension *N*_*E*_ × *N*_*E*_ with elements {λ_β_}. This transformation implies that |Ψ⟩ can be expressed as a Schmidt decomposition:
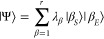
20where |β_*S*_⟩ = *∑*_*i*_*U*_*i*,β_|*i*⟩, , and *r* = min(*N*_*S*_, *N*_*E*_).^38^ {λ_β_} are called the Schmidt coefficients. It follows that in the Schmidt basis, the reduced density operator  can be written as
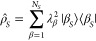
21where |β_*S*_⟩ and λ_β_^2^ = ω_β_ are the eigenstates and eigenvalues, respectively, of the reduced density operator.

The quadratic norm *S*_2_ is given by
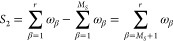
22*S*_2_ is therefore minimized by retaining the *M*_*S*_ eigenstates of  with the largest eigenvalues.

Once the truncated basis for the new system block of length *k*, {|*l*_*k*_⟩} is known, all operators, including the Hamiltonian, are rotated to the new Hilbert space via a similarity transformation.

**Figure 3:**
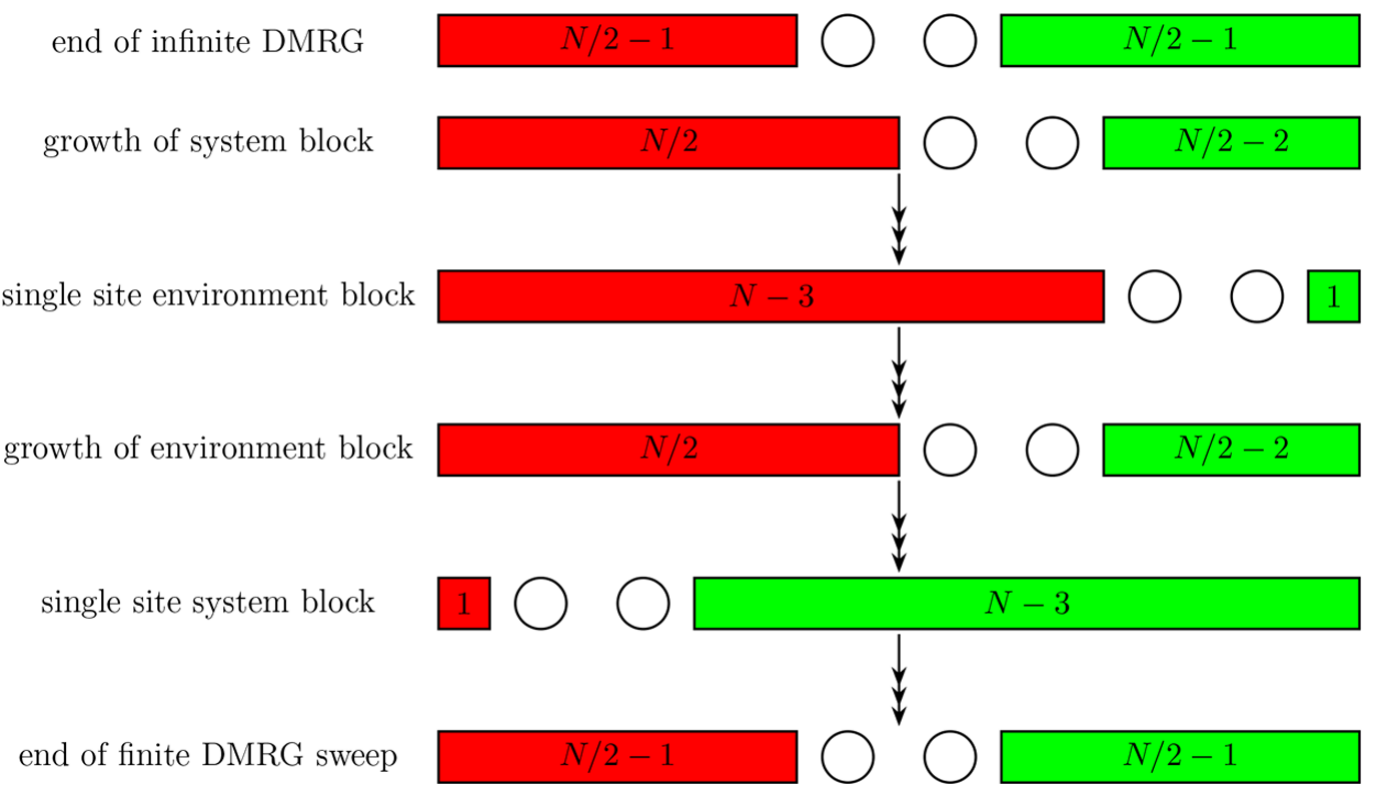
Schematic diagram for the finite DMRG algorithm illustrating a single finite lattice sweep.^79^ The number of sites in each block is shown as a text. An open circle represents a single site block.

The total Hamiltonian is not known during the intermediate steps of the infinite DMRG algorithm, and this leads to errors, especially in systems with strong physical effects from impurities or randomness in the Hamiltonian.^79^ These finite size effects can be resolved by performing finite “sweeps” after the infinite DMRG. Once the desired system size *N* is reached, the steps of infinite DMRG is continued, but with one block (system) growing at the expense of the other (environment). The superblock size remains fixed at *N*, and truncation of the basis is only performed for the growing block. Determination of the superblock ground state is efficiently implemented using the White’s wave function mapping algorithm,^80^ where the ground state found during the previous step of the sweep is rotated into the new Hilbert space to be used as a trial state for the diagonalization procedure. This procedure is continued until the shrinking block only contains a single site, and then the direction of the sweep is reversed. Several finite DMRG sweeps are performed until the desired convergence is reached. The finite DMRG algorithm is illustrated in Figure 3.

By exploiting the sparcity of the block symmetry operators (e.g., the particle-hole and spin-flip symmetries), excited states are conveniently determined by constructing symmetry-adapted states..^69^ Within a symmetry sector, higher-lying states are then determined via a Gram-Schmidt projection. In order to accurately describe these excited states, it is necessary to retain the basis states that optimally represent them in the truncated Hilbert space. This is achieved by including them in the reduced density matrix, i.e.
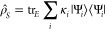
23where the summation includes all the targeted states. κ_*i*_ is usually chosen to be the same for all states, such that *∑*_*i*_κ_*i*_ = 1.

### 3.2 Adaptive Time-Dependent DMRG (tDMRG)

The dynamics of the evolving system under the Hamiltonian *Ĥ* is fully determined by solving the time-dependent Schrödinger equation:
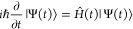
24In the limit that δ*t* → 0, eq 24 has the formal solution:
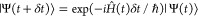
25

The adaptive time-dependent density matrix renormalization group method, developed in 2004, generalized the DMRG algorithm to study time dependent phenomena.^50,51^ In this formalism, the evolving state |Ψ(*t*)⟩ is determined in a truncated Hilbert space, such that the loss of information about the system is minimized. The algorithm is efficiently implemented for Hamiltonians containing only on-site and nearest neighbor interactions. Such a Hamiltonian can be written as a sum of bond Hamiltonians. By defining
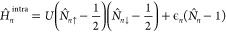
26and
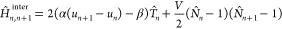
27we can write
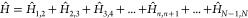
28where
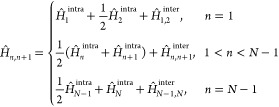
29acts on the *n*^th^ bond. Since neighboring bond Hamiltonians do not commute, a Suzuki–Trotter decomposition is invoked for the propagator, i.e.
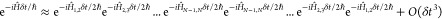
30

The link time evolution operator, , is exactly applied on |Ψ(*t*)⟩ at DMRG step (*n* – 1).^51^ At this step the DMRG state is
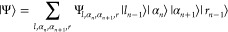
31The states |*l*_*n*–1_⟩ and |*r*_*n*–1_⟩ are the basis states corresponding to the system and environment DMRG blocks at step (*n* – 1). The states |α_*n*_⟩ and |α_*n*+1_⟩ are the exact basis states for sites *n* and *n* + 1.

To find , the 2-site augmented block state |*m*⟩ = |α_*n*_⟩|α_*n*+1_⟩ is transformed to the basis spanned by the eigenstates of , i.e., , where
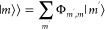
32With this transformation,  can be written as
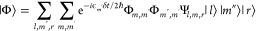
33

**Figure 4:**
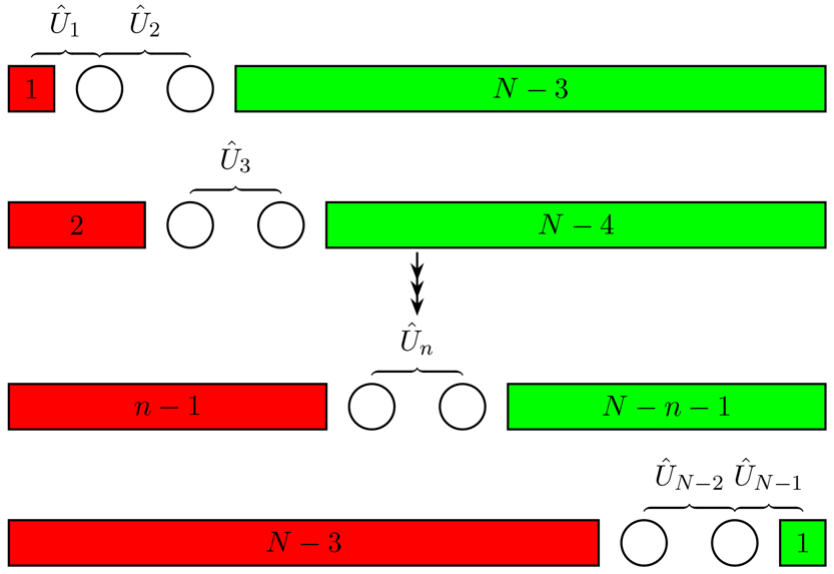
Schematic diagram for a single sweep of the adaptive tDMRG algorithm, illustrating the application of the time evolution operator for a time of Δ*t*/2. The number of sites in each block is shown as text. An open circle represents a single site block. .

The algorithm now proceeds in precisely the same way as for the static finite lattice DMRG method, namely |Φ⟩ is truncated via a singular value decomposition and is then transformed to the basis for the next DMRG step via White’s wave function mapping technique.^80^Figure 4 illustrates the key steps of the adaptive tDMRG algorithm.

Readers are referred to refs (79) and (81) for a review of the DMRG, refs (82−84) for recent reviews of applications of DMRG in quantum chemistry, and ref (85) for a review of time-dependent density matrix renormalization group methods.

### 3.3 DMRG Accuracy

As discussed in section 3.1, the DMRG algorithm finds the optimum  by minimizing the quadratic norm *S*_2_ (see eq 22). The truncation error, ϵ, associated with the DMRG algorithm can therefore be defined as the sum of the eigenvalues of  discarded during the DMRG truncation, i.e.
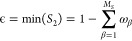
34and ∑_β=1_^*r*^ω_β_ = 1. The reason for the remarkable success of DMRG in explaining the properties of one-dimensional quantum systems is understood by the realization that the DMRG truncation error is closely related to the amount of information that is required to accurately represent a quantum system.^86,87^ This amount of information is dependent on the entanglement of the system and is quantified via the von Neumann entanglement entropy:
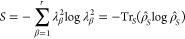
35

To illustrate this quantity, consider a fully unentangled system. This state is described by a product state given by
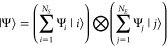
36It follows that for this state,  where , and thus *S* = 0. Therefore, the unentangled state has a minimum von Neumann entropy.

Recall from eq 20 that a general state |Ψ⟩ can be written using a Schmidt basis, where the Schmidt coefficients λ_β_ are related to the eigenvalues of  by ω_β_ = λ_β_^2^. Thus, if the eigenvalues {ω_β_} decay rapidly, then only a small error is introduced by retaining only a relatively small number of eigenstates.

The growth of entanglement with systems size is related to the “area law”, which explains why DMRG works so well for one-dimensional systems but fails in higher dimensions.^88^ The area law states that the entanglement entropy of the ground state of a gapped, partitioned system is proportional to the size of the partition between the systems. For one-dimensional systems, the surface between two systems is a point, and as this does not change with system size, the entanglement entropy is a constant with system size.^89^ This further implies that the truncation error remains essentially constant for a fixed system basis size as a function of the system size. DMRG is thus highly suitable for the study of the electronic states of insulating polyene systems.

## 4 Nuclear Degrees of Freedom

While the electronic wave function determined by solving the TDSE is achieved using adaptive tDMRG, the dynamics of the nuclei are determined via the Ehrenfest equations of motion. The Ehrenfest method assumes that the nuclei evolve on a single effective potential energy surface corresponding to an average of the electronic states contributing to the electronic wave function. As a result, despite its mean-field nature, the Ehrenfest method can describe transitions between different electronic states.^90^

The nuclear degrees of freedom, defined by eq 2, are treated classically via the Hellmann–Feynman theorem. The force on atom *n*, *f*_*n*_, is given by eq 14:
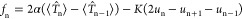
37The nuclei obey Newton’s equations of motion
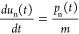
38and
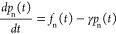
39where *p*_n_ and *m* are the nuclear momentum of the atom n and mass, respectively. A phenomenological linear damping term *γp*_n_ is introduced to cause relaxation of the nuclei. (The electronic energy gaps and the relevant phonon energy (=0.2 eV) are all much larger than the Boltzmann scale factor *k*_B_*T*. In addition, the dynamics are so fast that equilibration is not possible in relevant time scales. Therefore, except for damping, other temperature effects are neglected.) The equations of motion are propagated using the damped velocity Verlet scheme shown below:
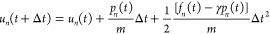
40
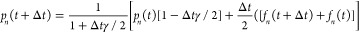
41

The changes in the nuclear displacements cause a change in the bond lengths, which in turn, change the Hamiltonian parameters, {β_*n*_}, as defined by eq 3.

**Figure 5:**
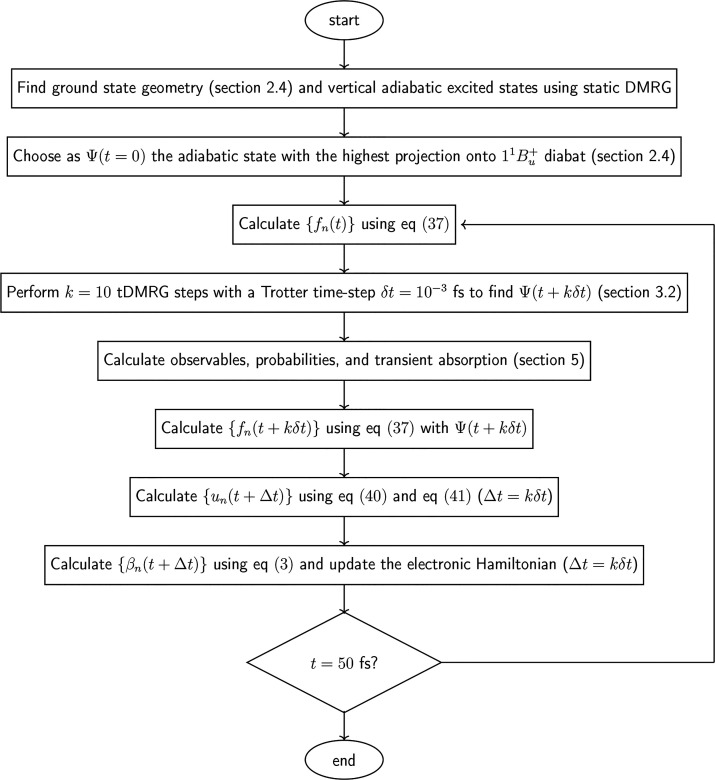
Schematic diagram illustrating the dynamical simulation scheme developed in this paper.

A schematic diagram illustrating the dynamical algorithm developed in this work is shown in Figure 5.

## 5 Lanczos-DMRG

Transient (time-resolved) absorption experiments are among the most routinely utilized experimental techniques in the field of carotenoid photophysics.^91^ A typical transient absorption measurement involves photoperturbation of the dynamical system at time *t* and measurement of the effect of the perturbation at time *t* + τ. This corresponds to the dynamical correlation function  defined as
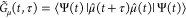
42where μ̂(*t*) is the Heisenberg representation of the dipole moment operator μ̂. Via a Fourier transform of ω with respect to τ, we obtain the dynamical correlation function in the frequency domain as^92^
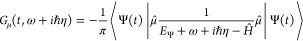
43where *E*_Ψ_ is the energy of |Ψ(*t*)⟩, and η is a small positive real number used to shift the poles of *G*_μ_(*t*, ω) into the complex plane. The imaginary part of *G*_μ_(*t*, ω + *i*ℏη) is given by
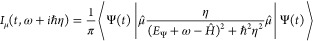
44If a complete set of eigenstates {|*n*⟩} of Ĥ, with eigenvalues {*E*_*n*_} is known, using , we can write
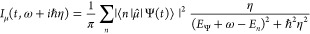
45In the limit η → 0, we obtain the transient absorption spectrum at time *t* via
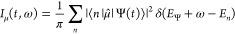
46

It is possible to calculate the transient absorption via a direct evaluation of {|*n*⟩} by targeting the *n* eigenstates in the DMRG density matrix.^93^ However, for a fixed number of retained DMRG density matrix eigenstates, the truncation error grows rapidly with the number of targeted states in the density matrix and thus limits the use of this approach. Hallberg^94^ and Kühner and White^95^ introduced the Lanczos-DMRG method which combines DMRG with the Lanczos algorithm.^96^

The Lanczos method was developed as an efficient algorithm to diagonalize matrices by transforming a given matrix to a special basis, where it is tridiagonal.^97^ Tridiagonal matrices are sufficiently sparse to be efficiently diagonalized using standard matrix diagonalization libraries. The special basis to which the Hamiltonian is transformed is spanned by Lanczos vectors {|*f*_*n*_⟩}. If *n* < *D*, where *D* is the dimension of the original matrix, then the matrix is said to be projected on to the Krylov subspace spanned by the Lanczos vectors. Lanczos-DMRG is based on the observation that since only a few eigenstates of the Hamiltonian make a finite contribution to the spectrum (as shown by eq 46), the original Hamiltonian can be projected on to a Krylov subspace of much smaller dimension which can accurately describe these excitations.

We calculate transient spectra from the state |Ψ(*t*)⟩ = |*S*_*i*_⟩ using the Lanczos-DMRG method, with normalized Lanczos vectors defined as in ref (95):
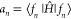
47
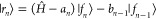
48
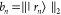
49and
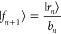
50where *b*_–1_ = 0. We require |*S*_*i*_⟩ to be the lowest energy eigenstate of the Hamiltonian in the projected Krylov subspace. This is achieved by projecting out the lower energy eigenstates via
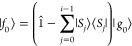
51where
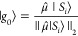
52Projecting out the eigenstates lower in energy than |*S*_*i*_⟩ is also motivated as we are only interested in the calculation of stimulated absorption spectra. By defining |*g*_0_⟩ in this way, we make sure the Krylov subspace captures the information about the excitations that make a finite contribution to the spectra accurately. (By choosing |*g*_0_⟩ to be a random state and *n* = *D*, any Hamiltonian can be transformed to a tridiagonal matrix using Lanczos diagonalization. See ref (38).)

We now outline the implementation of Lanczos-DMRG within our adaptive tDMRG simulations. Once the dynamical simulation for time *t* is reached, we perform a static DMRG sweep to calculate observables, ending at the DMRG step, where the system block is the same size as the environment block. We save the full Hilbert space, which is in the tDMRG basis. Then we perform a full static DMRG sweep, diagonalizing the Hamiltonian at each DMRG step to find |*S*_*i*_⟩, and the Lanczos vector {|*f*_*n*_⟩, as defined above. The first five Lanczos vectors are used as target states during the static DMRG sweep, weighted proportionately by their contribution to the spectrum as defined by eq 17 of ref (95):
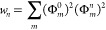
53where *w*_*n*_ is the weight of the Lanczos state *n*, , and |Φ_*n*_⟩ is the *n*th eigenstate of the Hamiltonian in the Lanczos Hilbert space. Noting that we need an accurate representation of all eigenstates up to and including |*S*_*i*_⟩ during the static DMRG sweep, we assign 25% of the weight to |Ψ(*t*)⟩, 25% of the weight to all eigenstates up to |*S*_*i*_⟩ (distributed equally), and the remaining 50% of the weight to the target Lanczos states. At the end of the static DMRG sweep, we calculate the transient absorption spectrum using eq 46. The dynamical simulation is then continued from the saved tDMRG Hilbert space.

Lanczos-DMRG is a simple and efficient method for the calculation of the low energy discrete absorption spectra and is well suited for a tDMRG simulation where the transient absorption is calculated as a function of time during the dynamics. More accurate, but expensive, frequency-space DMRG methods, such as correction vector DMRG^95^ and dynamical DMRG,^98^ are required for the calculation of complex spectra with high energy absorptions, or continuous spectra.

## 6 Accuracy and Convergence

The adaptive tDMRG algorithm utilized in the calculations of this paper and its companion^71^ suffer from two sources of error. First, the accuracy of the DMRG algorithm is dictated by the truncation error, defined by eq 34. This error is minimized by retaining more states during truncation. Second, the Suzuki-Trotter decomposition introduces the Trotter error which is reduced by reducing the Trotter time step, δ*t*. Minimizing the Trotter time step also minimizes the error associated with the use of a velocity Verlet integrator for the nuclear dynamics.

### 6.1 Truncation Error and Entanglement Entropy

Due to the variational nature of the DMRG algorithm, its accuracy can be systematically improved by increasing the number of states retained during the DMRG truncation step. Convergence of the closely related PPPP model has been extensively studied.^40,42,68,69^

**Figure 6:**
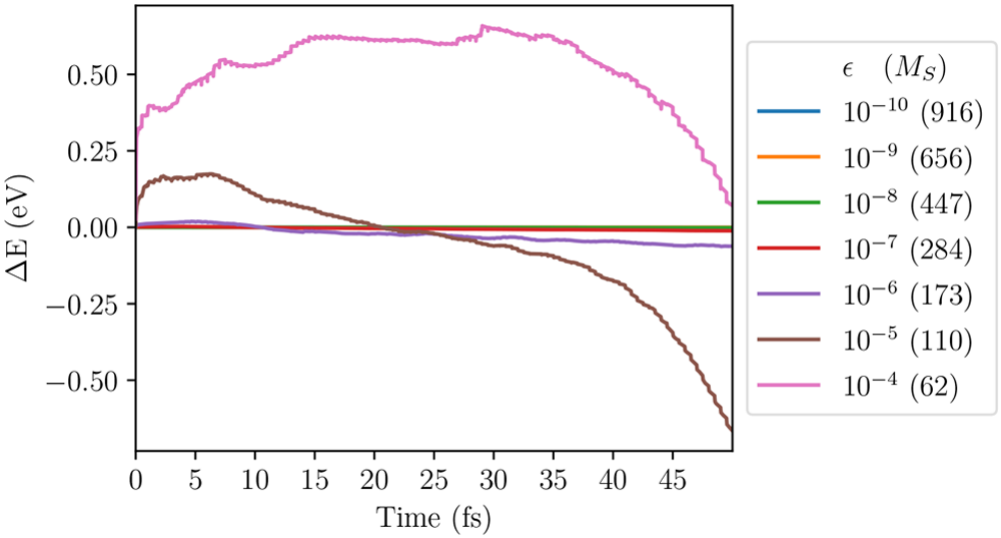
Calculated difference of the energy as a function of time of the system described by the evolving state vector |Ψ(*t*)⟩ for different DMRG truncation errors, ϵ, with respect to the energy calculated with a DMRG truncation error of 10^–11^. The maximum number of augmented block states, *M*_S_, retained to reach the specified DMRG accuracy is indicated within parentheses. The evolving state energy is converged for a DMRG truncation error of ϵ = 10^–8^.

The convergence of the DMRG truncation error can be determined by evaluating the convergence of an observable as a function of the truncation error. As an illustrative example, Figure 6 shows the variation of energy of the evolving wave function |Ψ(*t*)⟩ with the DMRG truncation error for neurosporene (*N* = 18), with *V* = 3.25 eV. The energy is converged for a truncation error of ∼10^–8^ to an accuracy of 0.1 eV.

In general, the number of states required to be retained in a DMRG scheme is given by^38^
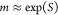
54where *S* is the von Neumann entanglement entropy (defined in eq 35). For our simulations, the maximum von Neumann entanglement entropy reached, *S*_max_ < 4.0, and thus from eq 54 the number of states required to retain *m* ≈ 55. The truncation cutoff of ∼10^–8^ is reached by typically retaining ∼400 augmented block states during the DMRG truncation, which is much larger than the number of states required by eq 54.

### 6.2 Trotter Error

**Figure 7:**
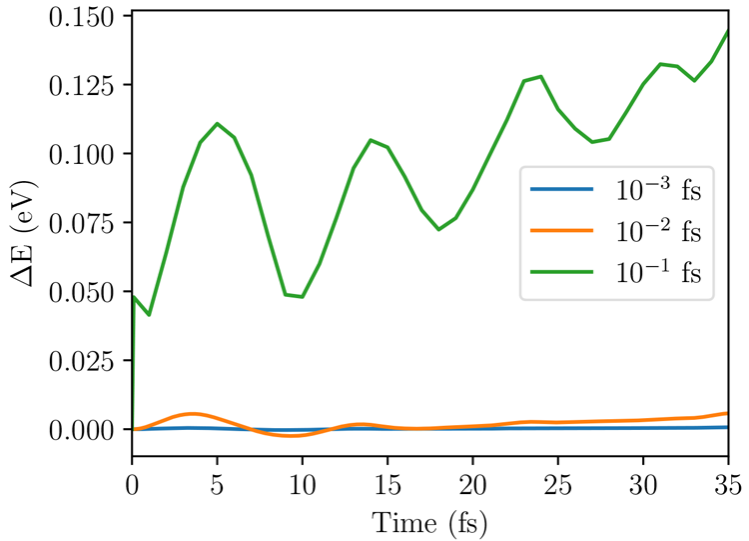
Calculated difference of the energy as a function of time of the system described by the evolving state vector |Ψ(*t*)⟩ for different Trotter time steps, δ*t*, with respect to the energy calculated with a Trotter time step of 10^–4^ fs. Convergence is reached with a Trotter time step of 10^–3^ fs.

For an accurate representation of nuclear dynamics, the time step Δ*t* should be much smaller than the time scale of nuclear motion, set by carbon–carbon bond oscillations. Typical carbon–carbon bond oscillation frequencies are ∼20 fs, and thus we require Δ*t* < 20 × 10^–3^ fs. Figure 7 illustrates the calculated expectation value of energy of the evolving wave function |Ψ(*t*)⟩ as a function of the Trotter time step δ*t*, for neurosporene (*N* = 18), with *V* = 3.25 eV. We notice that the energy is converged for δ*t* = 10^–3^ fs, and we use this value as the Trotter time step. We perform 10 tDMRG steps per each nuclear position update and therefore Δ*t* = 10^–2^ fs.

### 6.3 Accuracy of Lanczos-DMRG Calculations

**Figure 8:**
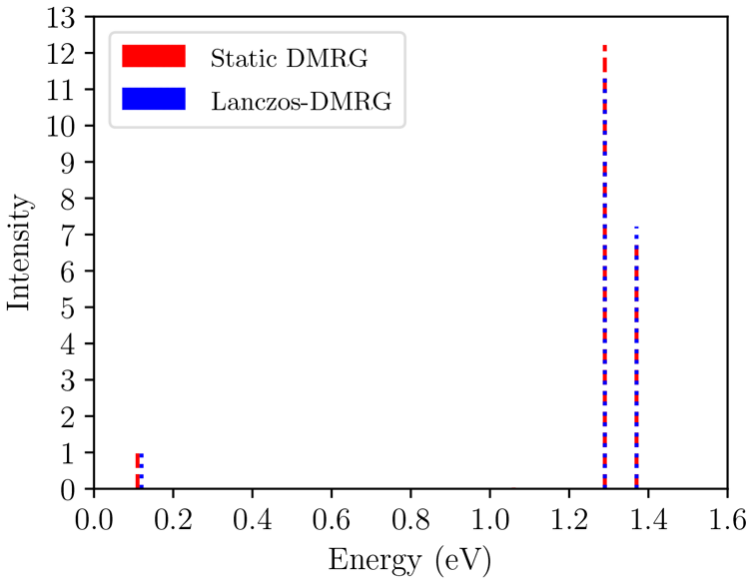
Transient absorption spectra calculated from the 1^1^B_u_^+^ state using static DMRG (red) and Lanczos-DMRG (blue). The three signals correspond to transitions to the 2^1^A_g_^–^, 4^1^A_g_^–^, and 5^1^A_g_^–^ states in order of increasing energy. The intensity (I) is normalized with respect to the 2^1^A_g_^–^ signal, i.e., *I*(*n*^1^A_g_^–^) = .

The 1^1^B_u_^+^ state is connected to the excited states in the A_g_^–^ sector by the dipole moment operator. Therefore, the accuracy of the Lanczos-DMRG can be evaluated by comparing the calculated transient spectra to the energies and transition dipole moments of the excited states in the A_g_^–^ sector, calculated using static DMRG in the absence of symmetry-breaking and in the vertical geometry. The results are shown in Figure 8, and they demonstrate a good agreement between the two methods.

Although targeting several Lanczos states during the static DMRG sweep of the Lanczos-DMRG procedure increases the accuracy of the calculation, it leads to a large computational expense. In our simulations, we target five Lanczos vectors, which maintain the DMRG truncation error at around ϵ ∼ 10^–8^ while keeping *M*_*S*_ ∼ 1000 augmented block states.

## 7 Approximate Two-Level Dynamics

### 7.1 Quasi-stationary State Dynamics

**Figure 9:**
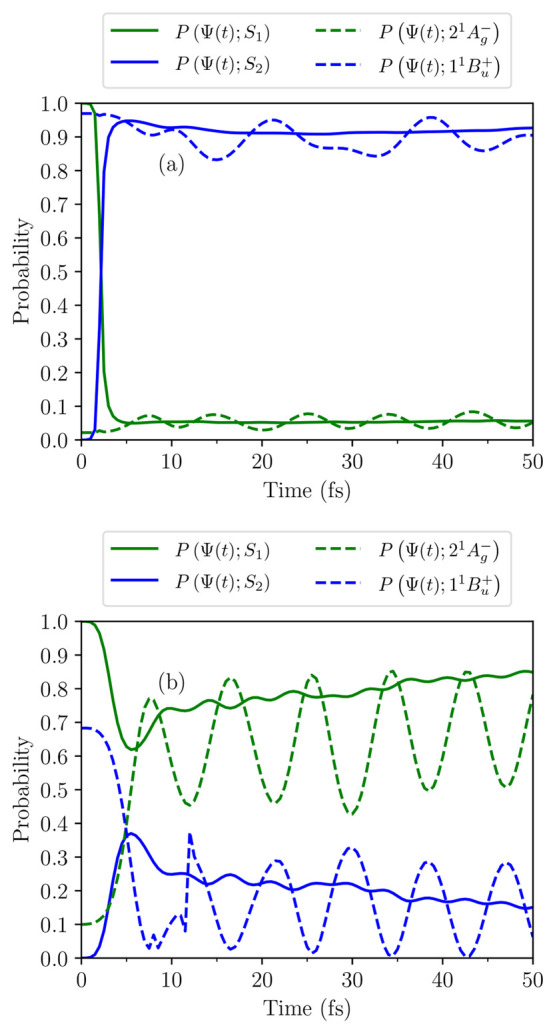
Probabilities as a function of time that the system described by Ψ(*t*) occupies the adiabatic states, *S*_1_ and *S*_2_ (calculated using eq 9), and the diabatic states, 2^1^A_g_^–^ and 1^1^B_u_^+^ (calculated using eq 10). The initial condition is that Ψ(0) = *S*_1_. Results are for *N* = 18 and *V* = 3.25 eV, and for the symmetry-breaking Hamiltonian presented in Table S1 of ref (71) by a linear scaling factor of (a) ζ = 0.2 (small symmetry-breaking) and (b) ζ = 1.2 (large symmetry-breaking).

As observed in ref (70), for molecules possessing *C*_2_ symmetry, the optically prepared state |Ψ(*t*)⟩ is almost entirely composed of two adiabatic states during the entirety of the time evolution. Thus, to a good approximation, we can adopt a two-level system and express |Ψ(*t*)⟩ as the nonstationary state
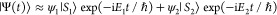
55where |*S*_1_⟩ and |*S*_2_⟩ are the two contributing adiabatic states, and the probability amplitudes ψ_1_ and ψ_2_ are assumed to be constant. Denoting the two diabatic states as |ϕ_1_⟩ and |ϕ_2_⟩, we can write (in an exact two-level system, |*a*|^2^ = |*d*|^2^ and |*c*|^2^ = |*b*|^2^):
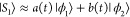
56and
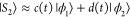
57Thus, the probability that the system occupies the diabatic state |ϕ_2_⟩, *P*(Ψ(*t*); ϕ_2_) = |⟨ϕ_2_|Ψ(*t*)⟩|^2^, is
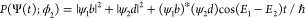
58Equation 58 describes the observed oscillatory behavior of the diabatic probabilities of the two-level system (as shown in Figure 9b). Omitting the oscillatory term, we define the “classical” probability of |ϕ_2_⟩ as
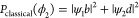
59i.e.
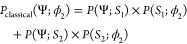
60

### 7.2 Landau–Zener-like Transition

In the absence of the particle-hole symmetry-breaking term, , a system prepared in the 1^1^B_u_^+^ state will remain on the same diabatic surface. In this limit, defined by ϵ_max_ → 0, the diabatic and adiabatic states are equivalent. Since the diabatic surfaces cross during the dynamical process, this implies that Ψ(*t*) will undergo a nonadiabatic transition from *S*_1_ to *S*_2_ while remaining in the 1^1^B_u_^+^ diabatic state; i.e., at the crossing the adiabatic states swap their ordering.

The inclusion of a nonzero , however, causes an avoided crossing of the adiabatic states. As illustrated in Figure 9a, for small symmetry-breaking, there is a nonadiabatic transition of Ψ(*t*) from *S*_1_ to *S*_2_, while Ψ(*t*) predominately occupies the 1^1^B_u_^+^ diabatic state. Conversely, as shown in Figure 9b, for large symmetry-breaking, there is an adiabatic transition: Ψ(*t*) predominately occupies *S*_1_, while there is a transition from the 1^1^B_u_^+^ to the 2^1^A_g_^–^ diabatic states.

The transition from a “fast” nonadiabatic process to a “slow” adiabatic process as a function of the symmetry-breaking parameter, ϵ_max_, corresponds to the classic Landau–Zener transition of a two-level system.^99−101^ In both cases, although the diabatic surfaces cross, the adiabatic surfaces exhibit an avoided crossing and not a conical intersection. We study this transition in more detail by linearly scaling the potential energies in the symmetry-breaking Hamiltonian (presented in Table S1 of ref (71)) by a scaling factor ζ.

**Figure 10:**
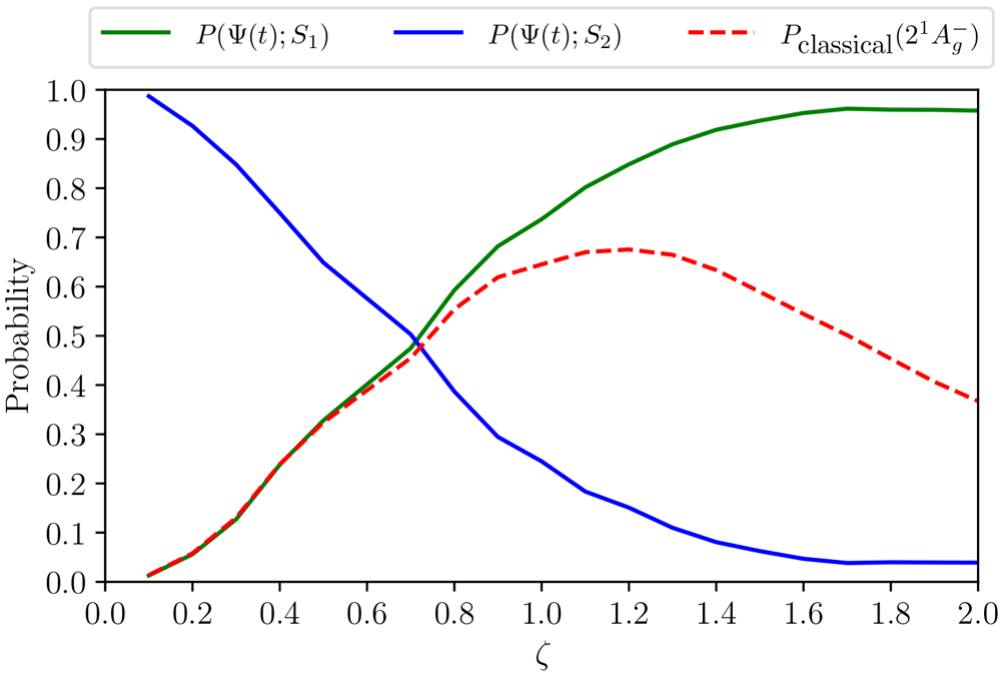
Probabilities at *t* = 50 fs as a function of increasing the strength of the particle-hole symmetry term (i.e., ζ) that the system described by Ψ(*t*) occupies the adiabatic states *S*_1_ and *S*_2_. Also shown is the classical 2^1^A_g_^–^ yield. The initial condition is that Ψ(0) = *S*_1_. Results are given for *N* = 18 and *V* = 3.25 eV.

Figure 10 illustrates the probabilities that the system described by Ψ(*t*) occupies the adiabatic excited states *S*_1_ and *S*_2_ at *t* = 50 fs as a function of ζ, given the initial condition that Ψ(*t* = 0) = *S*_1_. The probabilities *P*(Ψ; *S*_1_) and *P*(Ψ; *S*_2_) as functions of ζ illustrate the crossover from the nonadiabatic to adiabatic limits described above.

The “classical” probability, defined by eq 60:
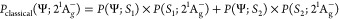
61provides a measure of the adiabaticity of the transition. We see that as the particle-hole symmetry-breaking term increases (i.e., as ζ increases), *P*_classical_ gradually increases and reaches a maximum ∼65% around ζ = 1.2. As ζ further increases, *P*(Ψ(*t*); 1^1^B_u_^+^) + *P*(Ψ(*t*); 2^1^A_g_^–^) < 1, i.e., *S*_1_ gains contributions from higher energy diabatic states. Consequently, the process can no longer be modeled as a two-level system.

For the relevant carotenoid parameters, described in ref (71), ζ = 1 and thus for carotenoids the internal conversion from the “bright” state to the “dark” state is essentially an adiabatic process.

## 8 Conclusions

This paper presents a dynamical simulation scheme to model the highly correlated excited state dynamics of linear polyenes. It complements our more explanatory discussion of carotenoid excited state dynamics in refs (70) and (71). We applied it to investigate the internal conversion processes of carotenoids following photoexcitation. We use the extended Hubbard-Peierls model, , to describe the π-electronic system coupled to nuclear degrees of freedom. This is supplemented by a Hamiltonian, , that explicitly breaks both the particle-hole and two-fold rotation symmetries of idealized carotenoid structures. The electronic degrees of freedom are treated quantum mechanically by solving the time-dependent Schrödinger equation using the adaptive tDMRG method, while nuclear dynamics are treated via the Ehrenfest equations of motion. By defining adiabatic excited states as the eigenstates of the full Hamiltonian , and diabatic excited states as eigenstates of , we present a computational framework to monitor the internal conversion process from the initial photoexcited 1^1^B_u_^+^ state to the singlet triplet-pair states of carotenoids. We further incorporate Lanczos-DMRG to the tDMRG-Ehrenfest method to calculate transient absorption spectra from the evolving photoexcited state. We describe in detail the accuracy and convergence criteria for DMRG, and show that this method accurately describes the dynamical processes of carotenoid excited states. We also discuss the effect of the symmetry-breaking term, , on the internal conversion process, and show that its effect on the extent of internal conversion can be described by a Landau–Zener-type transition.

We conclude by noting that with the correct model parametrization DMRG methods are highly suited to study the photodynamics of other low-dimensional π-conjugated systems, such as retinals,^102−108^ light-emitting polymers,^68,69^ and acenes (when considered as a set of coupled linear chains, see Figure 1 of ref (109)). As this list encompasses a large class of interesting and technologically important conjugated molecules, it implies that DMRG methods have a wide-range of applicability. Indeed, since these molecules are also highly correlated electron systems, DMRG methods are some of the very few computational methods that can accurately describe them.

The DMRG codes were developed by the Barford group and are available on request.
